# Profiles of host immune impairment in *Plasmodium* and SARS-CoV-2 infections

**DOI:** 10.1016/j.heliyon.2022.e11744

**Published:** 2022-11-18

**Authors:** Rini Chaturvedi, Mradul Mohan, Sanjeev Kumar, Anmol Chandele, Amit Sharma

**Affiliations:** a Molecular Medicine Group, International Center for Genetic Engineering and Biotechnology, New Delhi, Delhi, India; b Parasite-Host Biology Group, National Institute of Malaria Research, New Delhi, Delhi, India; c Academy of Scientific and Innovative Research (AcSIR), Ghaziabad, 201002, India; d ICGEB-Emory Vaccine Program, International Center for Genetic Engineering and Biotechnology, New Delhi, Delhi, India

**Keywords:** SARS-CoV-2, COVID-19, *Plasmodium*, Malaria, Cytokine storm, Immune impairment

## Abstract

Over the past two decades, many countries have reported a steady decline in reported cases of malaria, and a few countries, like China, have been declared malaria-free by the World Health Organization. In 2020 the number of deaths from malaria has declined since 2000. The COVID-19 pandemic has adversely affected overall public health efforts and thus it is feasible that there might be a resurgence of malaria. COVID-19 and malaria share some similarities in the immune responses of the patient and these two diseases also share overlapping early symptoms such as fever, headache, nausea, and muscle pain/fatigue. In the absence of early diagnostics, there can be a misdiagnosis of the infection(s) that can pose additional challenges due to delayed treatment. In both SARS-CoV-2 and Plasmodium infections, there is a rapid release of cytokines/chemokines that play a key role in disease pathophysiology. In this review, we have discussed the cytokine/chemokine storm observed during COVID-19 and malaria. We observed that: (1) the severity in malaria and COVID-19 is likely a consequence primarily of an uncontrolled ‘cytokine storm’; (2) five pro-inflammatory cytokines (IL-6, IL-10, TNF-α, type I IFN, and IFN-γ) are significantly increased in severe/critically ill patients in both diseases; (3) Plasmodium and SARS-CoV-2 share some similar clinical manifestations and thus may result in fatal consequences if misdiagnosed during onset.

## 1 Malaria and COVID-19: implications in co-endemic regions

The coronavirus-19 (COVID-19) pandemic has now crossed >530 million cumulative confirmed infections and ∼6.3 million deaths have been reported globally. However, at the time of this work, a decline in daily-confirmed COVID-19 cases is being reported [1]. Effective and specific antiviral drugs are still being developed against severe acute respiratory syndrome coronavirus-2 (SARS-CoV-2) [2]. COVID-19 vaccines have been deployed in multiple nations, and their efficacies are high, but data on immune longevity and protection is still being elucidated, specifically to evolving variants and sub-lineages. Compounded with the pandemic is the threat of spread or resurgence of other endemic and non-endemic diseases as the medical and public health systems across the globe were overwhelmed or disrupted due to COVID-19. Malaria is one such disease whose elimination programs may be affected in malaria-endemic countries due to the COVID-19 pandemic [3, 4, 5].

Malaria is caused by a mosquito-borne parasite of the Plasmodium species and was responsible for ∼241 million cases and 627,000 deaths in 2020 [6]. The disease is common in tropical countries of Africa and Asia. *Plasmodium falciparum* (Pf) is considered to be the deadliest Plasmodium species infecting humans and is the dominant species responsible for >95% of the estimated malaria cases globally. *P. vivax* (Pv) has the ability to form hypnozoites in the liver; if left untreated, Pv infection can relapse as it continues to persist in the liver. India (together with nineteen other sub-Saharan African countries) is responsible for 85 percent of the global malaria load and was severely afflicted by COVID-19, reporting the second-highest COVID-19 cases tally worldwide [1]. African countries were also affected by COVID-19 and reported >9.1 million cases with 0.17 million deaths [7]. India, with the second highest COVID-19 cases currently had a lower first wave caseload, with >90% positive cases being asymptomatic during the first peak [8].

Often malaria cases are asymptomatic and may contribute to malaria transmission in the regions [9, 10]. The Global Fund to fight AIDS, Tuberculosis, and Malaria reported that in 2020 malaria diagnosis and treatment fell by 56% and 59% in seven countries from Asia, while 17% and 15% in Africa, respectively [11]. In resource-limited areas where molecular diagnosis for SARS-CoV-2 is not readily available, early treatment may rely on symptomatic assessment with early symptoms of fever, cough, malaise, and headache. These symptoms are similar for COVID-19 and malaria; hence, misdiagnosis can occur in malaria-endemic regions [7]. This can exacerbate the problem of disease burden and may result in delayed diagnosis and inappropriate treatment [5]. Malaria has been shown to have severe adverse effects concurrent with other infections [12]. For e.g., Pf infections have been shown to increase HIV viral load, posing a higher threat to the co-infected host [12, 13]. Further, emerging reports of coinfection of malaria and Covid-19 highlight the association with increased all-cause in-hospital mortality compared to mono-infection with SARS-CoV-2 [14, 15]. SARS-CoV-2 severity is a continuum and disease symptoms can range from asymptomatic to mild to more severe forms of respiratory distress and organ failure. We still do not fully understand the repercussions of COVID-19 in areas already endemic to malaria [16, 17]. However, our understanding of the qualitative nature of the human immune response to SARS-CoV-2 and Plasmodium in individuals living in malaria endemic areas is evolving. This review aims to understand the profiles of overlapping cytokines/chemokines during human infection of SARS-CoV-2 or Plasmodium.

## 2 Shared symptoms of COVID-19 and malaria

During Pf infection, the parasite reaches the blood very quickly, triggering a cascade of immune responses in the human host. SARS-CoV-2 primarily enters through the respiratory route and affects the lungs most as they are also a highly vascularized organ. Both infections eventually result in systemic immune responses [18]. The severity of the disease by SARS-CoV-2 is suggested to be aggravated in cases with multiple comorbidities [19]. Both malaria and COVID-19 share similar early clinical manifestations (fever, fatigue, headaches of acute onset), while severe manifestation includes acute respiratory distress syndrome (ARDS) that might lead to multi-organ failure in critical patients [18, 20]. Given the similarity in early symptoms of COVID-19 and malaria, the possibility of misdiagnosis of a malaria patient as COVID-19 positive is higher and vice versa during malaria transmission seasons in the absence of quick and rapid diagnosis for COVID-19 and/or malaria. This is of particular concern in resource-limited settings, which are primarily areas that are also endemic to malaria.

In high-burden regions of malaria, mortality is associated with infants, children and pregnant women [21]. It was originally considered that COVID-19 infections result in asymptomatic or mild recoverable symptoms in children and severe symptoms in the elderly population predominantly [22, 23]. However, newer epidemiological studies suggest increased vulnerability in younger age groups [24]. Though milder symptoms in children can be multi-factorial, one of the recognized reasons is a more robust innate immune response that is the frontline defense mechanism, especially to viral infections [25]. Another reason could potentially be the pediatric immune system is prepared to react and defend against novel pathogens as natural antibodies (IgM isotype produced by IgM memory B cells) play important roles in the early phase of infections and have broad reactivity that can prevent reinfection [26]. This property of fending off novel pathogens slowly diminishes in the elderly as age-associated immunosenescence sets in [26]. Furthermore, B cells (neonatal and activated) along with IgA-producing antibody-secreting plasmablasts, are responsible for secreting the potent anti-inflammatory cytokine interleukin (IL)-10 in hosts [27]. A Ffew outcomes of severe Pf malaria are cerebral malaria (CM), severe anemia, and malaria-associated ARDS (MA-ARDS). One of the reasons for severe malaria in patients can be attributed to misdiagnosis or delayed diagnosis of malaria. ARDS is also one outcome of SARS-CoV-2 [28]. If left untreated, these disease outcomes can lead to death. The repercussions of misdiagnosis due to common early symptoms or delayed or incorrect treatment may allow both diseases to progress towards ARDS, which can be subsequently fatal. It is noted that in malaria infections, African children suffer mostly from severe anemia [29, 30]. At the same time, other complications like ARDS and acute kidney injury (AKI) may predominate mostly in adolescents and adults [30, 31].

## 3 Comparison of the cytokine surge in malaria and COVID-19

**Figure 1:**
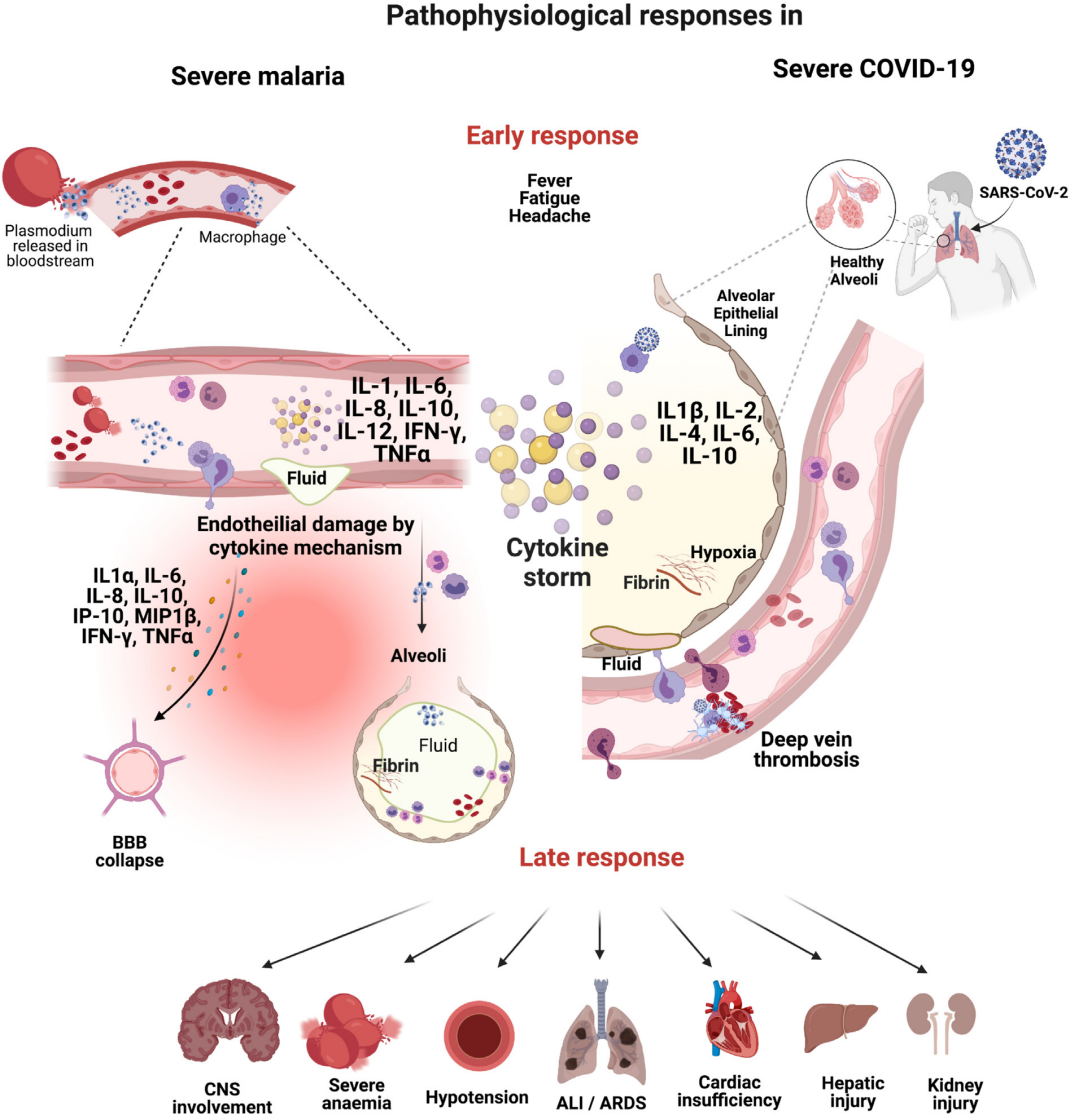
The figure illustrates the clinical symptoms and the cytokines involved in dysregulation of the pathophysiology in severe COVID-19 and malaria infections. The image has been created with BioRender.com.

When a body encounters a pathogen, the first line of defense is the innate immune response. The specialized myeloid and lymphoid cells sense the entry of a pathogen through pattern recognition receptors (PRRs) like retinoic acid-inducible gene 1 (RIG-1)-like receptors (RLR), nucleotide-binding oligomerization domain (NOD)-Leucine Rich Repeats (LRR)-containing receptors (NLR), C-type lectin receptors (CLR), toll-like receptors (TLRs), etc. [32]. These PPRs sense pathogen-associated molecular patterns (PAMPS) and trigger downstream signaling pathways that ultimately result in the release of cytokines/chemokines that set off the downstream immune cascade of both cellular innate cells and adaptive immune responses [32]. These immunological effects are also associated with a cytokine/chemokine signature that can be either protective or not [33]. When the host cells get infected, innate immune responses are triggered through various pattern recognition systems – TLR/NLR/NOD-like receptors. This eventually results in the further presentation of cognate antigen to cells of the adaptive immune response that, in turn, produce cytokines such as interleukin-6 (IL-6), IL-12, type I and II interferons (IFNs), and tumor necrosis factor (TNF), etc. Both B and T cells are also known to produce chemokines that allow the immune cascade to continue. Regulated & controlled production of cytokines/chemokines eventually results in pathogen clearance with little to no pathology, whereas uncontrolled secretion of cytokines/chemokines by either the innate or adaptive arm commonly referred to as ‘cytokine storm’ can be detrimental to the host cell [34, 35]. It is now established that the severity of the disease is not only the outcome of invasion and multiplication of the pathogen but also may be a manifestation of an unwarranted or heightened cytokine storm that can be induced during infection, and severe symptoms can occur after the pathogen is cleared [36]. Infection can stimulate high-level activation of immune cells leading to excessive production of chemical mediators and inflammatory cytokines to combat the invaded pathogen. The surge of almost 20 different inflammatory cytokines/chemokines triggers the activation of various types of immune cells resulting in excessive inflammation. Uncontrolled inflammation occurs due to the secretion and presence of inflammatory cytokines at the site of infections. This leads to clumping of cells that try to clear the pathogen and pathogen-infected cells. Inflammation is a multifactorial event, which occurrs at the advent of foreign molecule invasion, which initiates the activation of various immune cells. However, sometimes inflammation continues and does not decrease as the pathogen is cleared and may culminate in pathology [34]. These factors may vary in different diseases. One outcome may be ARDS that can lead to multi-organ failure and death. This cytokine storm and resultant immunopathology is a possibility in both COVID-19 and severe malaria (Figure 1) [35, 36, 37].

Both malaria and COVID-19 have documented surges in cytokine in patients (Table 1). While a cytokine storm occurs in both COVID and malaria, the profiles differ and this is going to be discussed in greater detail below. Earlier studies had indicated that severely/critically ill COVID-19 cases had elevated serum levels of interleukins (IL-1β, IL-2, IL-4, IL-6, IL-10) (Figure 1, Table 1) [38]. Among the interleukins, IL-6 – a pleiotropic, proinflammatory cytokine produced by several cells like lymphocytes, monocytes and fibroblasts, is of major significance owing to its pleiotropic effects, and it has been linked to COVID-19 severity [39]. Moreover, elevated levels of IL-6 have been associated with the severity of COVID-19, implying an ‘added push’ toward lung damage [39]; thus it has been considered as a biomarker of COVID-19 severity [40]. While IL-6 at homeostatic levels helps repair tissue injuries and infections, its increased production significantly accelerates cytokine storms [41, 42]. Consequently, anti-IL-6 monoclonal antibody (tocilizumab) therapy has been approved for the management of critically ill COVID-19 infections [43]. Baricitinib – an oral Janus kinase (JAK) inhibitor used for rheumatoid arthritis (RA) – is understood as an immunomodulator and it inhibits cytokines implicated in intensive care unit-bound COVID-19 patients [44]. It has been demonstrated that baricitinib inhibits human peripheral blood mononuclear cell production of monocyte chemoattractant protein-1 (MCP-1) stimulated by IL-6 cytokine [45]. Baricitinib's anti-inflammatory benefits have also been shown in pediatric patients with steroid-dependent chronic inflammation via a decrease in serum levels of interferon-γ (IFN-γ), interferon γ-induced protein (IP-10), granulocyte macrophage colony-stimulating factor (GM-CSF), and MCP-1 [46]. Given these factors, baricitinib is also being investigated to assess the beneficial effects on COVID-19 patients similarly to tocilizumab [47]. Similarly, sarilumab – a human monoclonal antibody initially used for RA acting against IL-6 - is also under consideration for the treatment of COVID-19 if tocilizumab is not available [47].

**Table 1:** Data published on the upregulated or downregulated cytokines in the patients afflicted by COVID-19 or malaria due to Pf/Pv. The upward single arrows show the elevated levels while the single arrow pointing down depicts the decreased levels of the cytokines/chemokines or the CD4/8 + T cells. Double upward arrows depict significantly increased levels of cytokines and T cells while double downward arrows depict significantly decreased levels of cytokines and T cells in both diseases. IL stands for interleukins, GM-CSF – Granulocyte macrophage colony-stimulating factor, G-CSF – Granulocyte colony-stimulating factor, IP – interferon γ-induced protein, MCP-1 – monocyte chemoattractant protein-1, MIP1α – macrophage inflammatory proteins α, MIP1β – macrophage inflammatory proteins β, TNF – tumor necrosis factor, IFN – interferon.

Cytokines/Chemokines	COVID-19		*Pf* Malaria		Cerebral Malaria	*Pv* Malaria	
	Mild	Severe	Blood stage/mild	Liver stage		Primary Malaria	Recurrent
IL-1			↑		↑		
IL-1β		↑↑	↑				
IL-2		↑↑	↑			↑	
IL-4	↑		↑			↑	↑↑
IL-5			↑				
IL-6	↑	↑↑	↑↑		↑↑	↑	↑↑
IL-7	↑	↑↑					
IL-8	↑	↑	↑↑				
IL-10	↑	↑↑	↑↑		↑↑	↑	↑↑
IL-12			↑↑	↓	↑		
IL-21			↑				
GM-CSF			↑↑				
G-CSF	↑						
IP-10	↑	↑↑			↑↑		
MCP-1		↑↑	↑↑				
MIP1α	↑	↑↑	↑↑		↑		
MIP1β			↑↑		↑↑		
TNF-α	↑	↑↑	↑↑	↓	↑↑	↑	
IFN-γ	↑	↑↑	↑↑	↓	↑↑	↑	
TypeI IFN	↓	↑↑	↑	↑			
CD4+T cells	↓	↓↓	↑	↓		↓	↓↓
CD8+T cells	↓	↓↓		↑	↑↑	↓	↓↓
Lymphotoxin					↑↑		

Another interleukin of interest is IL-10, which has been shown to increase prior to IL-6 [48,49]. IL-10 is generally regarded as ‘anti-inflammatory and immune inhibitory’ stimulated by accumulating proinflammatory cytokines as a negative feedback mechanism [50, 51]. However, elevated IL-10 levels are associated with the severity of viral infections such as dengue [52]. IL-10 has been reported to be dramatically elevated in serum of severe/critical COVID-19 [38, 50, 53]. Recent research suggests that high IL-10 levels in severe COVID-19 patients indicate immunological activation and inflammation, implying that IL-10 may play a role in proinflammatory and immune-activating responses in COVID-19 pathogenesis [51, 54].

In malaria, a number of cytokines are induced in both the liver and blood stages [55, 56]. In mouse models of liver-stage malaria, there is a distinct increase in IL-4, IL-10, TGF-β, and downregulation of IFN-γ, IL-12, and TNFα [55, 57, 58]. On the other hand, symptomatic malaria occurs during the blood stages when merozoites are released in the bloodstream and results in a cascade of inflammatory cytokines such as IL-6, IL-8, IL-10, IL-1, and IL-12 that are associated with a “cytokine storm” that is implicated in disease severity (Figure 1, Table 1) [59]. It is also suggested that, like COVID-19, severity of blood-stage malaria correlates with the circulating IL-6 levels [40, 60, 61, 62]. Thus, both diseases show overlapping cytokine signatures associated with disease outcomes. Previous studies have reported that IL1α, IL-8, IL-6, and IL-10 are particularly elevated in serum of children suffering from cerebral malaria compared to those with severe anemia [63, 64]. This cytokine signature is similar to those that several studies have reported for COVID-19 infections [35, 37, 63, 64, 65, 66]. Further, circulating concentrations of IL-6 are increased in proinflammatory critical care syndromes, such as sepsis and ARDS [67]. Therefore, the use of the IL-6 type of biomarker, which is now used for monitoring disease prognosis in COVID-19 patients, should be revisited as a prognostic marker in malaria-endemic regions.

Another family of cytokines, the type I interferons (most studied are IFN-α and IFN-β), along with tumor necrosis factor (TNF-α), are considered important for antiparasitic, antiviral, and bacterial defense [68, 69, 70]. These cytokines, specifically type I interferons, are rapidly triggered after recognition of viral nucleic acid by host sensors such as PAMPs and have a range of direct and indirect effects ranging from inhibition of viral replication to the recruitment and activation of various immune cell types [71, 72]. Though type I interferons are necessary and are the first line of defense for viral clearance, their overproduction or systemic/uncontrolled production can cause immunopathology and immunosuppression during acute and chronic viral infections, respectively [73]. SARS-CoV-2 was previously thought to be a poor inducer of type I IFNs [74, 75]. However, there is increasing evidence that patients have robust type I IFN responses leading to activation of neutrophils in severe COVID-19 infections [76, 77, 78]. The transcriptomic studies revealed an elevated expression of interferon-stimulated genes (ISGs) in classical monocytes in the lungs of severe COVID-19 patients [76, 77]. The proinflammatory action of type I IFNs were also shown in murine models of SARS-CoV-2 and reported to recruit proinflammatory monocytes and macrophages to the infected lungs [79]. It is a possibility that the type I IFNs might be contributing to the hyper inflammation responses by intensifying the inflammation driven by TNF/IL-1β in the severe progression of COVID-19 [76]. In SARS-CoV-2 infections, there is a delayed and deregulated IFN release. This delay in IFN release contributes to low levels of IFN-I in the early stages and higher levels in the advanced stages of COVID-19 [80, 81]. Several SARS-CoV-2 proteins can inhibit IFN-I and IFN-II production. As a result, patients succumb to COVID-19 early due to hyperinflammation, contributing to the early lack of IFN responses to SARS-CoV-2 [80]. It is hypothesized that the early IFN response might control the viral replication while delayed response might lead to pathological inflammation in COVID-19 [76]. The upregulation of proinflammatory ISGs might lead to either protection from the virus by amplification of the inflammatory signals to the lungs or may lead to deleterious pathological inflammation, especially when a plethora of cytokines and other immune responses have already been triggered [76, 78]. The confounding nature of type I IFNs might be explained by differences in severity of the disease and the time point of sampling [76]. Interestingly, though malaria is a parasitic disease, upregulation of type I IFNs has been reported in both liver and blood-stage infections [55, 82]. In the liver stages of murine models of malaria, the transcriptomic studies revealed type I IFN signaling recruits leukocytes to eliminate parasites in hepatocytes [55, 83].

IFNs have shown to be effective in suppressing Plasmodium parasitemia with improved host survivability and they enhance humoral immunity with stimulation of dendritic cells to promote isotype switching [84]. Alternatively, IFNs are also reported to contribute to immune suppression and promote the production of inflammatory cytokines/chemokines causing severity in and pathogenesis of the experimental cerebral malaria [84, 85]. Type I IFNs are involved in modulating dendritic cells function leading to impairment in IFN-γ producing parasitic-specific CD4+T cells, thus promoting the development of IL-10-producing T regulatory (Treg) cells [86]. It has been observed that if high levels of type I IFNs are produced early in the infections (24 h post-infection and later downregulated to the basal level), they acquire a protective role in blood stages of malaria [84]. However, the failure in downregulation of IFNs leads to chronically elevated levels which then causes immune suppression in malaria infection [84]. Another interferon IFN-γ (a type II interferon) is a key checkpoint regulator for many cytokines [87]. IFN-γ operates partially by activating STAT1 signalling, but mostly by inducing gp130 internalization, a common component of multiple heterodimeric cytokine receptors, including IL-6 [87]. IFN-γ is induced in both COVID-19 [88] and malaria [89] and is important for downstream cellular responses. In viral infections, the role of IFN-γ secreted by natural killer cells and antigen-presenting cells has been implicated in early host defense and autocrine regulation. Once the adaptive immune response is triggered, the T cells are the major source of IFN-γ. IFN-γ activates macrophages and can illicit immune responses like enhanced antigen processing and presentation, increased reactive oxygen species production, autophagy induction and increased secretion of proinflammatory cytokines – all these are necessary processes for clearance of pathogens of any origin, be it viral or parasitic. Hence, it is not surprising that the absence of IFN-γ responses is associated with increased susceptibility to both viral and parasitic diseases [90].

Tumor necrosis factor-alpha (TNFα) expressed by various innate and adaptive immune cells is a multifunctional proinflammatory cytokine that is robustly expressed and associated with severe malaria, as it is with severe COVID-19 [38,91]. In malaria, TNFα is produced during both the pre-erythrocytic and erythrocytic stages and inhibits parasite growth [92, 93, 94]. However, the spikes in TNFα levels in serum have also been shown to correspond with elevated body temperature in Pv patients (Table 1) [93]. Raised TNFα levels in blood and the brain have also been reported in the patients who succumbed to CM [95]. TNFα increases the expression of cell adhesion molecules such as ICAM-1 and E-selectin on the endothelium of the vascular surface, causing infected RBCs to attach to the microvasculature and eventually causing blockage of blood vessels [96, 97, 98, 99]. TNFα also induces the activation of lymphocytes and these hyper-activated cells create immune pathology at the site of infection [97, 98]. In SARS-CoV-2 infections, TNFα plays a critical role in enhancing cytotoxicity that leads to lung tissue injury [100]. In cytokine storm, the high concentrations of TNF causes negative regulatory effect on T-cell survivability and proliferations [100]. Reports of IFN-γ and TNFα sensitizing the cells to initiate inflammatory cell death in lung cells have been documented in COVID-19 [91]. This results in damage of lung capillary endothelial and alveolar epithelial cells, thus causing an increase in permeability of the lung microvascular system wherein fibrin-rich fluid is exuded to lungs leading to edema in the lungs [35, 100, 101]. This ‘capillary leak’ is a major determinant of acute lung injury (ALI) that leads to ARDS [100, 102]. The respiratory failure caused by the cytokine storm, which causes ARDS, is one cause of death due to COVID-19 [103].

It is to be noted that the multi-organ involvement found in severe malaria is not a simple consequence of an altered cytokine profile. Other factors such as cytoadherence of parasitized erythrocytes to endothelial cells, formation of clumps in microvascular in major organs, and production of reactive nitrogen intermediates with free heme-mediated reactive oxygen intermediates also play crucial roles [104, 105, 106]. Similarly, SARS-CoV-2 infection triggers a wide range of immuno-inflammatory, vascular thrombotic, and parenchyma derangements that lead to dysregulation in host response [107]. The inflammation in the endothelium and microthrombi formation in alveoli is followed by an increased perfusion in the lung, or dead space ventilation, causing a rise in diffusion barrier leading to impaired lung function and tissue hypoxia [107]. These mechanisms hence may together contribute to the severity of COVID-19 infections [107].

## 4 Concluding remarks

Multiple pro-inflammatory cytokines are raised during severe malaria and COVID-19 infection. In particular, five pro-inflammatory cytokines (IL-6, IL-10, type I IFNs, IFN-γ, and TNF-α) are significantly increased in severely/critically ill patients. These cytokine signatures are similar though one stems from a viral infection and another from a parasite. The uncontrolled release of IL-6, IFNγ, and TNF-α can result in a downstream cascade of cytokine and cellular responses that further contribute to disease severity in both COVID-19 and malaria. Our work highlights the nature of immune impairment observed during these two diseases. Exploration of therapeutic strategies that target the dampening of the cytokine storm can provide novel avenues for the treatment of COVID-19 and malaria.

## 5 Search strategy and selection criteria

References for this review were identified through searches of PubMed and BioRXiv for articles published from January 1990, to April 2021, by use of the terms “COVID-19”, “SARS-CoV-2”, “Plasmodium”, “malaria”, “cytokines”, “chemokines”, “cytokine storm”, and “vaccination”. Articles resulting from these searches and relevant references cited in those articles were reviewed and added to the current review. Articles published only in English were included.

## Declarations

### Author contribution statement

All authors listed have significantly contributed to the development and the writing of this article.

### Funding statement

This research did not receive any specific grant from funding agencies in the public, commercial, or not-for-profit sectors.

### Data availability statement

Data included in article/supp. material/referenced in article.

### Declaration of interest's statement

The authors declare no conflict of interest.

### Additional information

No additional information is available for this paper.
